# Intra- and Extra-Cellular Events Related to Altered Glycosylation of MUC1 Promote Chronic Inflammation, Tumor Progression, Invasion, and Metastasis

**DOI:** 10.3390/biom6040039

**Published:** 2016-10-13

**Authors:** Sandra Cascio, Olivera J. Finn

**Affiliations:** 1 Department of Immunology, University of Pittsburgh, Pittsburgh, PA 15261, USA; 2 Fondazione Ri.Med, via Bandiera 11, Palermo 90133, Italy

**Keywords:** mucin 1 (MUC1), protein-protein interaction, tumor microenvironment, inflammation

## Abstract

Altered glycosylation of mucin 1 (MUC1) on tumor cells compared to normal epithelial cells was previously identified as an important antigenic modification recognized by the immune system in the process of tumor immunosurveillance. This tumor form of MUC1 is considered a viable target for cancer immunotherapy. The importance of altered MUC1 glycosylation extends also to its role as a promoter of chronic inflammatory conditions that lead to malignant transformation and cancer progression. We review here what is known about the role of specific cancer-associated glycans on MUC1 in protein-protein interactions and intracellular signaling in cancer cells and in their adhesion to each other and the tumor stroma. The tumor form of MUC1 also creates a different landscape of inflammatory cells in the tumor microenvironment by controlling the recruitment of inflammatory cells, establishing specific interactions with dendritic cells (DCs) and macrophages, and facilitating tumor escape from the immune system. Through multiple types of short glycans simultaneously present in tumors, MUC1 acquires multiple oncogenic properties that control tumor development, progression, and metastasis at different steps of the process of carcinogenesis.

## 1. Introduction

Mucin 1 (MUC1) is a heterodimeric type I transmembrane glycoprotein expressed on the apical surface of most epithelia, including mammary gland, lung, pancreas, kidney, female reproductive tract, and stomach. Like other mucins, MUC1 normally provides a physical barrier that protects luminal surfaces of most glandular and ductal epithelial tissues. The extended *O*-glycan branches on MUC1 form a mucinous gel that maintains cell surfaces lubricated and hydrated, protecting from external changes of pH and limiting the access of bacteria, microbes, and degradative enzymes. 

Various inflammatory premalignant and malignant conditions are associated with alterations in MUC1 expression and glycosylation that impact its cellular localization and function. Mucin glycosylation aberrations affect a variety of cellular activities, including growth, differentiation, and transformation. In addition, altered mucins affect adhesion, invasion, and immune surveillance.

**Figure 1:**
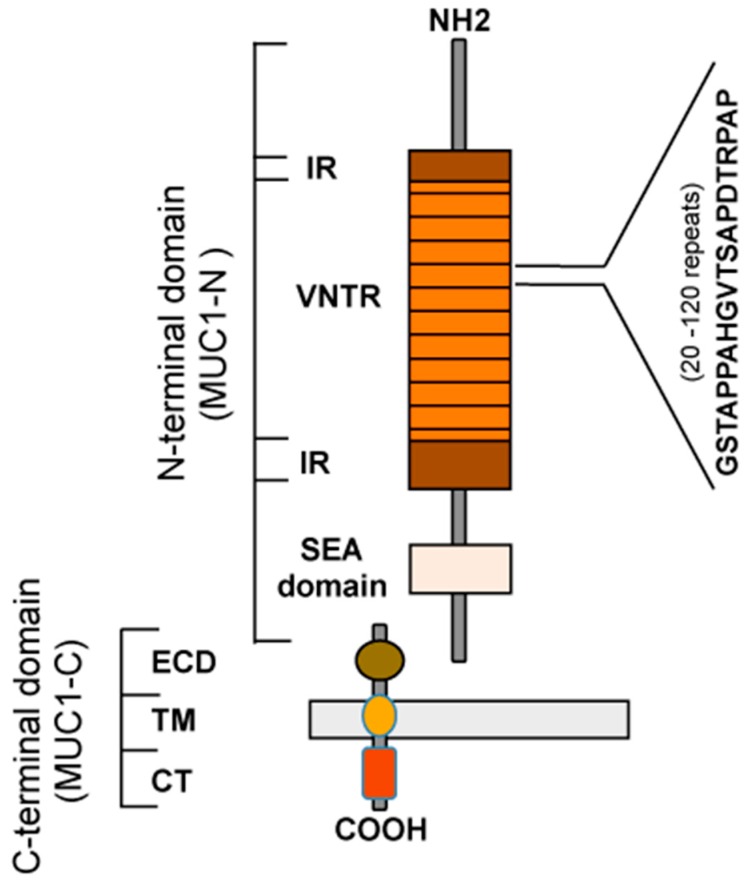
Schematic representation of mucin 1 (MUC1) structure. Abbrevition: CT: cytoplasmic tail; ECD: extracellular domain; IR: imperfect repeat; TM: transmembrane; SEA: sea urchin sperm protein-enterokinase-agrin; VNTR: variable number of tandem repeat.

Structurally, the full-length form of MUC1 consists of two subunits: a long N-terminal fragment (MUC1-N or subunit α), and a short C-terminal fragment (MUC1-C or subunit β) bound by non-covalent interactions (Figure 1). The MUC1-N contains the variable number of tandem repeats (VNTR) region and the SEA (sea urchin sperm protein enterokinase and agrin) domain. The VNTR is composed of a 20-amino acid (aa) peptide sequence (PDTRPAPGSTAPPAHGVTSA) repeated nearly perfectly between 20–200 times per molecule, rich in proline, threonine, and serine residues (PTS domain). Proline residues provide rigidity and contribute to a highly extended protein structure, while threonines and serines serve as sites for extensive *O*-linked glycosylation. The VNTR is flanked by five proximal and two distal imperfect repeats (IR) which contain 4–9 serine/threonine residues each and are probable additional glycosylation sites [1]. The SEA domain contains the proteolytic cleavage site, GSVVV-containing module, that generates MUC1 subunits α and β soon after its translation as a single protein. Some amino acid sequences in the SEA are also important for the non-covalent association of protein subunits (Figure 1). MUC1 subunits remain associated through a hydrogen bond, but they can be released from the cell surface by additional proteolytic cleavage event. The cleavage and release of membrane-tethered mucins are implicated in the pathogenesis of many inflammatory conditions and cancers including pancreatic, ovarian, and breast carcinomas [2,3,4]. 

The ectodomain shedding event is a very common and highly regulated cellular process that modulates the functional properties of proteins [5] and regulates a number of biological processes such as signal transduction, adhesion, and proliferation [6]. The MUC1 heterodimer dissociation is induced by inflammatory stimuli such as interferon gamma (IFN-γ) and tumor necrosis factor alpha (TNF-α), through activation of specific enzymes including the matrix metalloprotease ADAM (a disintegrin and metalloprotease)-17 and TNF-α containing enzyme (TACE) [7]. In addition, the cleavage sites in the extracellular region are often found in close proximity to *O*-glycosylation sites and hence glycosylation may affect proteolytic processing of the protein backbone [8,9,10]. 

The MUC1 C-terminal subunit contains a short extracellular domain (ECD) of 58 aa, a transmembrane domain (TM) of 28 aa that anchors MUC1-N to the cell surface, and a 72-aa cytoplasmic tail (CT). MUC1-CT is highly conserved and contains phosphorylated tyrosines. Through CT phosphorylation, MUC1 activates and mediates intracellular signaling by associating with various protein partners and modulating their function [11]. In tumor cells, increased tyrosine phosphorylation of MUC1-CT leads to interactions with new proteins, some of which are also aberrantly expressed in cancer. Specific examples are ErbB growth factor receptor tyrosine kinases, β-catenin, and the estrogen receptor. Binding of MUC1 with the epidermal growth factor receptor (EGFR), Erb2, and Erb3 activates signaling proteins including growth factor receptor-bound protein 2 Grb2, son of sevenless (SOS) and mitogen-activated protein kinase (MAPK) [12]. Phospho-MUC1 has a lower affinity for glycogen synthase kinase 3 (GSK3), which increases its binding to β-catenin, leading to the activation of the Wnt signaling pathway [13]. MUC1 stimulates the estrogen receptor-mediated transcription by binding directly to the estrogen receptor DNA-binding domain [14]. Moreover, MUC1-CT is also shown to modulate activity of the nuclear factor kappa B (NF-κB) pathway in breast cancer cells by interacting with and activating IkB kinase (IKK) family members and NF-κB p65 [15,16,17]. These new interactions of MUC1 with various proteins increase its internalization and enable nuclear localization, leading to altered signaling in cancer cells. 

## 2. Aberrant Glycosylation of MUC1 Is Associated with Cancer

Altered glycosylation patterns are considered to be one of the hallmarks of cancer and represent one of the most common post-translational modifications that occur during neoplastic transformation. We have analyzed some of the mechanisms responsible for the effect of altered MUC1 glycosylation on the tumor microenvironment. *N*-glycans and *O*-glycans are two major groups of carbohydrates that contribute to many intra- and extra-cellular functions of MUC1. Both glycosylation types are regulated by complex mechanisms, including the expression and activity of glycosyltransferases.

### 2.1. *O*-glycosylation

**Figure 2:**
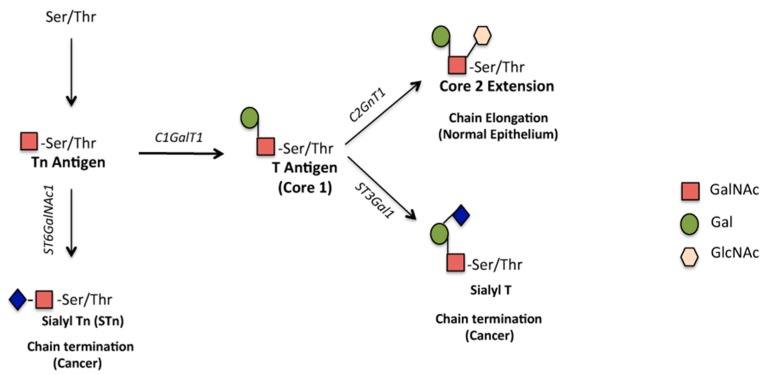
Schematic representation of *O*-Linked glycan biosynthesis

Mucin type *O*-glycosylation is initiated by polypeptide *N*-acetylgalactosaminyltransferase (GALNT) family members that add *N*-acetylgalactosamine (GalNAc) to either serines or threonines of PTS domains. The resulting *O*-linked structure is called Tn antigen. By the transfer of galactosamine (Gal) from UDP-Gal to GalNAc-α-1-R, the glycosyltransferase core 1 synthase, glycoprotein-*N*-acetylgalactosamine3-beta-galactosyltransferase, 1 (C1GalT1) (or T-synthase) generates the core 1 *O*-glycan, also named T antigen, which is the precursor of many extended *O*-glycans. In normal epithelial cells, the enzyme 2-β-1,6-*N*-acetylglucosaminyltransferase (C2GnT) elongates the core 1 *O*-glycan by the addition of GlcNAc (Figure 2). In cancer, the *O*-glycan chains attached to glycoproteins are often truncated and they commonly contain the Tn and T antigens, and their sialylated versions (STn and ST) [18,19]. The altered glycosylation occurring in tumor cells often results from aberrant expression of glycosyltransferases and their substrates that regulate their activity. Lack of C1GalT1 activity results in the exposure of Tn whereas the abundance of STn form is associated with aberrant overexpression of the α6-sialyltransferase ST6GalNAc1 that controls the sialylation of the first sugar GalNac of Core 1 (Figure 2). In breast cancer, the expression of RNA encoding ST6GalNAc1 correlates with sialyl-Tn expression [20]. Overexpression of the Tn form is also associated with somatic mutations in the core 1 β3-Gal-T-specific molecular chaperone, *COSMC*, gene that encodes a chaperone protein for the Core 1 synthase. The active form of C1GalT1 depends on the coexpression with *COSMC*, which prevents its aggregation and proteasomal degradation [21]. Depletion of the *COSMC* gene in vivo in mouse models causes loss of C1GalT1 activity and Tn antigen expression [22]. Epigenetic silencing of the *COSMC* gene, through promoter hypermethylation, occurs in prostate and other epithelial human cancers and correlates with overexpression of truncated *O*-glycans as well as C1GalT1 reduced expression [23]. Another enzyme often overexpressed in cancer is the α-2,3-sialyltransferase (ST3GalI) that adds sialic acid to core 1 glycans and is responsible for the abundance of the ST form (Figure 2). In breast carcinoma, ST3GalI expression is elevated when compared to normal or benign tissues and in ductal carcinoma the level of its messenger RNA (mRNA) is related to the grade of the tumor [24]. Overexpression of ST3GalI results in increased sialylation of MUC1 [25].

Underglycosylated forms of MUC1 are highly expressed in breast, colon, pancreas, and bladder tumors, among others, but not in normal tissues, and are often associated with epithelial to mesenchymal transition and invasiveness of cancer cells [26,27]. 

The role of STn has been investigated in breast cancer cells by inducing overexpression of α-*N*-acetylgalactosaminide-α-2,6-sialyltransferase 1 (ST6GalNAcI) enzyme. Clones of T47D breast cancer cell line overexpressing STn showed slower cell growth but increased cell migration. Moreover, adhesion of STn+ cells to several types of matrix proteins was decreased [28]. Transfections of ST6GalNAc1, performed in chinese hamster ovary (CHO) cell line, induced the production of MUC1 glycoform carrying STn epitope [20]. In MUC1 transgenic mice, sialyl-*O*-glycans have been shown to be associated with an enhanced growth of transplantable mammary carcinoma [29]. Overexpression of STn in human gastric cancer cell lines also accelerated their ability to form intraperitoneal metastases resulting in shorter survival of mice, credited mostly to MUC1 as the major STn carrier [30]. A recent evaluation of differential expression of mucin-associated glycans during colon carcinogenesis showed that MUC1-Tn and MUC1-STn are overexpressed in premalignant polyps, advanced adenoma, and adenocarcinoma, but not on the normal colon [31]. Both Tn and STn antigens are markers of poorly differentiated adenocarcinomas and mucinous carcinomas, and their increased occurrence is associated with highly proliferative tumors, metastasis, and poor clinical outcome [32,33]. Therefore, MUC1 STn and Tn forms are potential markers for early cancer diagnosis and prognosis and also targets for novel therapeutic strategies. 

In addition to T, Tn, and their sialylated forms, structural studies on *O*-linked glycans on the MUC1 repeat peptide revealed that other types of glycans are carried on cancer cells. For example, on ZR-75 and MDA-MB-231 breast cancer cell lines, MUC1 glycans include core 2-trisaccharides that terminate with 3-sialylated-galactose [34]. Mass spectrometric determination of MUC1-transfected prostate cancer cell line C4-2B_4_, revealed that the most prominent *O*-glycan on MUC1 was the non-sialylated and non-fucosylated core 2 oligosaccharide Galβ3/4GlcNAcβ6 [35]. However, small amounts of core 2 sialylated and fucosylated, as well as core 1 structures were also detected [35]. In gastric cancer tissues, a large variety of oligosaccharides have been detected, including both sialylated and fucosylated core 1 and 2 structures as well as large amount of oligosaccharides on MUC1 with α6-linked sialic acid, (NeuAcα6)GalNAc-ol and Galβ3(NeuAcα6)GalNAc-ol [36]. 

In ovarian, renal, prostate, and colon cancer, another type of shorter glycan structures associated with poor survival are MUC1 sialyl-Lewis^x^ (SLe^x^) and sialyl-Lewis^a^ (SLe^a^) [37,38,39,40,41]. SLe structures contain sialic acid and fucose and are derived by addition of β-1,4-galactose to form SLe^x^ and β-1,3-galactose to form SLe^a^ on core 2 structures. Metastatic cancer cells often have increased amounts of SLe^x^ and SLe^a^ when compared with primary tumors and expression of SLe^x^ on mucin *O*-glycans is highly associated with lymphatic and venous invasion [42,43,44]. SLe altered glycans serve as ligands for E- and P-selectins present on vascular endothelial cells. Therefore, cancer cells might use the SLe^x^-selectin binding mechanism to promote their extravasation and metastasis [45,46]. This hypothesis has been recently confirmed by Zhang et al. who showed evidences that SLe structures increase cell adhesion and confer an anti-apoptotic property to cancer cells leading to chemoresitance in ovarian cancer [47]. 

### 2.2. *N*-glycosylation 

While *O*-glycosylation has received extensive attention and mass spectrometry technology has enabled a detailed structural characterization of MUC1-related *O*-glycans, less is known about the *N*-glycosylation. *O*-linked glycans are attached to serines and threonines, while *N*-linked glycans are attached to the asparagine residue. MUC1 has five potential *N*-glycosylation sites: four are located in the C-terminal end of the VNTR, and the fifth is in the degenerate repeat flanking the VNTR [48,49].

Similar to *O*-glycosylation, *N*-glycosylation is important for the stability, folding, and secretion of glycoproteins. Membrane-derived and secreted-MUC1 showed a distinct *N*-glycosylation pattern [49], however the biological significance of this different glycan profile needs to be determined.

## 3. Aberrant Glycosylation of MUC1 Facilitates New Interactions with Cellular Proteins and Affects Intracellular Signaling in Cancer Cells

Carbohydrates covalently attached to proteins have been shown to play significant functional roles in proper protein folding, stability, and active biological conformation. Consequently, their alterations directly affect the properties of proteins, with important biological consequences for cellular differentiation, development, and progression of neoplasia and cancer immunosurveillance. Changes in carbohydrates also affect the physicochemical properties of proteins. Specifically, glycosylation modulates their thermostability as well as the overall charge. The addition of even a single monosaccharide to a protein can significantly impact the fluctuation in protein folded and unfolded states, enhancing its thermal and kinetic stability [50]. 

**Figure 3:**
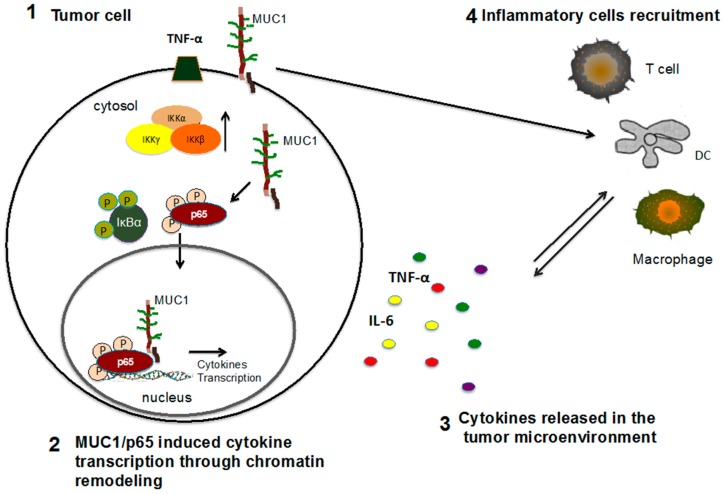
Model of MUC1 role in the tumor microenviroment. (**1**) Upon inflammatory stimuli, MUC1 induces activation of nuclear factor kappa B (NF-κB) members and binds phopsho-p65; (**2**) MUC1/phospho-p65 complex translocates to the nucleus and associates with the promoter of pro-inflammatory cytokines, such as interleukin 6 (IL-6) and tumor necrosis factor alpha (TNF-α), promoting their transcription and expression; (**3**) Secretion of inflammatory cytokines recruits inflammatory cells, including myeloid cells and neutrophils, into the tumor site; (**4**) Inflammatory cells are a further source for pro-inflammatory and pro-tumorigenic cytokines that enhance tumor growth and progression. DC: dendritic cells; IKK: IκB kinase; IκB: phospho-inhibitor of kappa B.

Changes in glycosylation have a significant impact on the physiological and pathophysiological activity of MUC1 as well. Loss or acquisition of glycans affects interactions of MUC1 with other cellular proteins. We recently demonstrated that cbl-interacting protein of 85 kDa (CIN85) is a MUC1-interacting protein implicated in migratory and metastatic activities of cancer cells. Notably, the interaction between MUC1 and CIN85 is dependent on the glycosylation state of the MUC1 extracellular domain. Overexpression of STn-MUC1 forms, induced in vitro by transfection of ST6GalNAc1, significantly enhanced the binding of MUC1 and CIN85 in breast cancer cells. Similar results were obtained after knocking-down the expression of C1GalT1, responsible for the conversion of Tn antigen to T antigen, which enhanced MUC1-CIN85 interaction [51]. The role of the extracellular domain of MUC1, and in particular its aberrant glycosylation, in facilitating new protein-protein interaction and controlling intracellular signaling was confirmed by another of our studies revealing the ability of the tumor form of MUC1 to increase the expression levels of NF-κB family members, such as phospho-IκB kinase (IKK)-β and phospho-inhibitor od kappa B (IκB)-α. Upon stimulation with TNF-α, the hypoglycosylated form of MUC1 accumulates in the cytosol in association with the activated form of p65 (phospho-65) and then translocates to the nucleus where it controls transcription of several pro-inflammatory cytokines (Figure 3) [17]. 

Another example of a key role of MUC1 glycosylation in protein-protein interactions is the interaction between galectin-3 and MUC1. Galectin-3 is a galactoside-binding protein expressed intra-and extra-cellularly as well as found free in circulation. Many studies have reported that the binding of galectin-3 with MUC1 triggers MUC1-dependent intracellular signaling in cancer cells and facilitates adhesion of cancer cells to each other and to endothelial cells [52]. Whereas there is an agreement on the function of this complex, opposing opinions have been reported about the binding domain of MUC1 to galectin-3. Ramasamy et al. indicated that galectin-3 binds the MUC1-C terminal subunit at the glycosylated Asn-36 site [53]. On the other hand, Yu et al. indicated that only the cancer-associated MUC1 is the natural ligand of galectin-3, this interaction being mediated through the T-antigen [54]. Yet another study showed that Galectin-3 binds MUC1-N terminal domain and this association triggers MUC1-mediated signaling, leading to the recruitment of β-catenin to MUC1-C [55].

Other evidence exists confirming the role of hypoglycosylated MUC1 in aberrant signaling in cancer cells. In breast cancer, overexpression of *N*-acetylgalactosaminyltransferase (GALNT6) contributes to increased proliferation possibly through stabilization of MUC1 protein. GALNT6-MUC1 pathway induces morphologic changes in cells accompanied by increased expression of cell adhesion molecules β-catenin and E-cadherin [56]. Another example is C1GalT1, an enzyme overexpressed in breast carcinoma that correlates with higher histological grade and advanced tumor stage. C1GalT1 also promotes breast cancer cell growth, migration, and invasion. It has been suggested that C1GalT1 enhances the malignant phenotype by inducing *O*-glycosylation changes on MUC1 and modulating the MUC1/β-catenin signaling pathway [57]. In inflammatory lung conditions induced by cigarette smoke, aberrant glycosylation of MUC1 promoted MUC1/p120 complex formation, as well as E-cadherin degradation and disassembly of adherence junctions, facilitating epithelial to mesenchymal transition (EMT) [58]. 

Changes in glycosylation impact not only the conformation of folded MUC1, its protein stability, and interaction with other proteins, but also its subcellular localization. Shorter *O*-glycans can slow down the delivery of MUC1 to the cell surface or increase MUC1 internalization from the surface to the cytosol, potentiating its oncogenic function [1,59]. 

## 4. MUC1 Glycosylation in Cancer Progression and Metastasis

Increased levels of MUC1 expression and its altered glycosylation have been associated with cancer progression and metastasis that correlate with poor prognosis and high mortality [3,60,61,62] Metastasis is a multi-step process during which cancer cells leave the primary tumor site, infiltrate into the adjacent tissue by degrading the extracellular matrix, enter into blood stream wherein they are recognized as circulating tumor cells, and extravasate and colonize a secondary site. 

The involvement of *N*- and/or *O*-linked glycans in cancer metastasis has been explored in vitro by transfections of cells with plasmids expressing specific glycosyltranferases, by addition of glycosylation sites, or by using drugs that affect oligosaccharide chain elongation. Results indicated an important role of aberrant glycosylation of MUC1 both at an early as well as the late stage of the metastasis cascade.

We recently demonstrated the important role of MUC1 in the early stage of metastasis. By associating with CIN85, an adaptor protein previously reported to be involved in cell migration [63], MUC1 confers an invasive property to cancer cells. Importantly, overexpression of the hypoglycosylated STn- and ST-MUC1 forms, induced by transfection with glycosyltransferases ST6GalNAc1 and ST3Gal1 encoding plasmids, enhanced its interaction with CIN85 and increased the migratory and invasive activities of cancer cells [51]. In addition, overexpression of ST6GalNAc1 that promotes STn type glycans on MUC1, increased intraperitoneal metastasis of human gastric cancer cells [30] and led to increased growth of human breast cancer cells in immunodeficient mice [64]. 

Recently, computational modeling revealed that bulky glycoproteins modulate transmembrane receptor spatial organization and function. In particular, MUC1 expression facilitates integrin clustering and focal adhesion assembly. By promoting focal adhesion assembly, MUC1 promotes also tumor cell growth and facilitates metastasis [65]. A critical step preceding metastasis is adhesion of circulating tumor cells to the vascular endothelium. Tumor-specific forms of MUC1 promote tumor cell adhesion to the endothelium through interactions with E-selectin and the intercellular adhesion molecule-1 (ICAM-1), two receptors expressed on the surface of endothelial and peritumoral stromal cells. The interaction between MUC1 and ICAM-1 facilitates cell-cell adhesion and trans-endothelial migration [66,67]. Moreover, upon interaction with ICAM-1, MUC1 initiates cytoskeletal rearrangement and shows increased pro-migratory activity [68]. As mentioned above, the extracellular Galectin-3 acts as an adhesion protein in cell-cell interactions and is involved in cancer progression and metastasis. Galectin-3 is highly expressed on circulating breast, colon, and lung cancer cells [69]. Patients with metastasis have higher concentration of circulating galectin-3 than patients with localized tumors, which binds the tumor specific carbohydrate T-antigen to promote metastasis [70].

Numerous other findings demonstrate the key role of abnormal MUC1 and related glycosyltransferases in tumor progression and metastasis. Recently, the polypeptide *N*-acetylgalactosaminyltransferase 3 (GALNT3) gene promoter was found to be significantly hypomethylated in epithelial ovarian cancers (EOC) compared to normal tissue. GALNT3 is overexpressed exclusively in high-grade serous ovarian tumors compared to tumors with low-malignant potential and normal tissues. Functional analyses revealed the oncogenic activity of GALNT3 in EOC, including its role in cell proliferation and cell migration and invasion. Knockdown of GALNT3 induced reduction of MUC1 expression in EOC cells although transcription of MUC1 was unchanged. Thus, GALNT3 may influence the posttranslational modification and stabilization of MUC1 and other glycosylated proteins in EOC cells [71]. In breast cancer cells, upregulation of GALNT6, a glycosyltrasferase that transfers a GalNAc residue to MUC1 protein, stabilizes MUC1 and plays a critical role in the proliferation and cytoskeletal regulation of breast cancer cells [56]. 

## 5. MUC1 Hypoglycosylation in Inflammation

In addition to cancer, changes in glycosylation are associated with both acute and chronic inflammation. The key molecules involved in adaptive and innate immunity are glycoproteins such as the major histocompatibility complex (MHC) class I and II. In addition, during maturation, the glycan chains of antigen receptors on B and T cells are modified with subsequent alteration of their cell surface molecular interactions that regulate receptor trafficking, signal transduction, and receptor internalization. Furthermore, alterations of immunoglobulin G (IgG) glycosylation have been linked with autoimmune diseases such as rheumathoid arthritis, Lambert-Eaton Syndrome, and Syogren’s Syndrome. In the review of Maverakis et al., it was postulated that different autoimmune diseases have a specific glycan profile characterized by the site-specific relative abundance of glycan structures expressed on immune cells and extracellular proteins [72]. 

The origin of altered glycosylation in cancer has been investigated by Haab’s group [73] Specifically, to test the hypothesis that pro-inflammatory stimuli affect mucin glycosylation, they stimulated six pancreatic cancer cell lines with IFN-γ, interleukin-1 (IL-1), and TNF-α and evaluated alterations in glycosylation and expression. Expression levels of MUC1, mucin 5 (MUC5), and mucin 16 (MUC16) increased with MUC1 showing the greatest change. They also calculated the glycans/protein ratio and their results suggested that glycans on mucins were remodeled in response to pro-inflammatory stimuli [73]. Pro-inflammatory cytokines TNF-α and IFN-γ also enhanced the expression of MUC1 in breast cancer cells through NF-κB and signal transducer and activator of transcription 1 (STAT1) activation, respectively [74]. It is also known that persistent activation of STAT3 signaling following epidermal growth factor (EGF) or IL-6 stimulation, results in upregulation of MUC1 (and other mucins) via direct binding of STAT3 to specific elements on the MUC1 promoter [75,76]. Exposure to IFN-γ and TNF-α increased MUC1 expression in ovarian cell lines that showed either high (cell lines OAW42 and GG) or low (cell lines JAM and PE01) basal expression of MUC1 [77]. Similarly, ocular epithelial cell lines increase expression of MUC1 after exposure to inflammatory mediators present in tears [78]. In oral epithelial cells, MUC1 expression is modulated by inflammatory mediators including IL-1, IL-6, TNF-α, and IFN-γ [79,80,81]. 

These findings support our ongoing experiments revealing overexpression of hypoglycosylated MUC1 in MDA-MB-231 breast cancer cells after TNF-α stimulation. Previously, we published that TNF-α treatment induced the association between hypoglycosylated form of MUC1 and NF-kB p65, the translocation of MUC1/p65 to the nucleus and further promotion of transcription and expression of inflammatory cytokines [17]. This mechanism establishes an autocrine and paracrine positive feedback loop that amplifies and maintains high levels of inflammatory cytokines in epithelial cancer cells and the tumor microenvironment (Figure 3). As TNF-α stimulation is responsible for increasing expression of β-galactoside-α-2,3-sialyltransferase 4 (ST3GalIV) and human alpha (1,3)-fucosyltransferase IV (FUTIV) through activation of NF-κB pathway [82], it is possible that pro-inflammatory cytokines influence mucin glycosylation through direct modulation of glycosyltransferase expression. However, the exact mechanism that links the inflammatory stimuli with altered glycans remains to be further explored. 

The link between aberrant pattern of MUC1 glycosylation and inflammation-related cancer has been extensively demonstrated in our laboratory also in in vivo models. We have used mice transgenic for the human MUC1 gene (MUC1.Tg) that express the full-length form of MUC1 in the same spatial and tissue distribution as the human protein [83]. The mouse MUC1 shares 87% homology in the cytoplasmic domain and only 34% homology in the extracellular domain of human MUC1. Thus, human MUC1.Tg mice represent a very good model to investigate the role of the human MUC1 extracellular domain and its altered glycosylation in inflammation and cancer development. 

It is well accepted that chronic inflammation increases the risk of cancer development and further enhances cancer progression. One example of inflammation-related cancer is colitis-associated cancer (CAC). A commonly used mouse model of CAC is based on administration of the combination of azoxymethane (AOM), a potent carcinogen, and the inflammatory agent dextran sulfate sodium (DSS). We found that after AOM/DSS treatment, the expression level of hypoglycosylated MUC1 was significantly increased in colon tissues. Furthermore, the presence of human MUC1 aggravated colitis symptoms, causing greater loss of body weight and higher mortality rate, significantly increasing the incidence of tumors [84,85]. 

IL-10 knockout mouse is another well-established mouse model of inflammatory bowel disease (IBD) wherein inflamed colon is characterized by inflammatory cell infiltration, crypt abscess formation, and epithelial hyperplasia. However, in only less than 20% of these mice, IBD progresses to adenocarcinoma. We demonstrated that introduction of human MUC1 into the IL-10 knockout mouse accelerated the occurrence of IBD, induced a more severe grade of inflammation, and promoted cancer development and progression in over 80% of the mice. Moreover, a drastic difference in the intensity of the cellular infiltrate between MUC1/IL-10 mice and IL-10 mice was detected. Importantly, hypoglycosylated form of MUC1 was detected at low levels in tissue sections with little inflammation whereas it was significantly increased in inflamed tissue, correlating with the inflammation score [86]. 

## 6. Abnormal MUC1 in the Tumor Microenvironment

The network of tumor cells and non-malignant cells creates the tumor microenvironment (TME). Non-malignant components of the TME include inflammatory cells, fibroblasts, and tumor vasculature and lymphatics. The composition of TME depends on the type of cancer, patient’s age, and other factors that still need to be characterized. Indeed, most successful anti-cancer treatments target not only the malignant cells, but also various components in the TME that promote cancer progression and inhibit anti-cancer immunity. 

In our mouse models of IBD and CAC described above [85,86], profound differences were detected in MUC1+ mice in pro-inflammatory cell populations at the sites of inflammation as well as in the tumor microenvironment. Specifically, in the presence of human MUC1, extensive accumulation of myeloid-derived suppressor cells (MDSC) dominates the spleen while they are absent in wild-type mice. The exact mechanism by which the tumor form of MUC1 modifies the tumor microenvironment is still unknown. Recent studies indicated that the interactions between altered glycans and lectins on immune cells are involved in the modification of the tumor microenvironment. For example, siglecs, members of the lectin family, are expressed on immune cells and have immunosuppressive properties. They recognize sialylated glycans [87,88] expressed on tumor cells. Co-culture of macrophages and lung cancer cells revealed a strong interaction between siglec-15, normally present on the surface of tumor associate macrophages (TAMs), and STn-glycans of cancer cells leading to production of TGF-β increased in Siglec15+ cells [89].

In breast cancer, MUC1 interacts with sialoadhesin (Sn), a receptor present on the majority of infiltrating macrophages that specifically binds the sialylated form of MUC1 [90]. Macrophages that play a key role in the adaptive and innate immune responses have been classified in two subpopulations: the classically activated macrophages (Type I), and the alternatively activated macrophages (Type II). Type 1 macrophages produce pro-inflammatory cytokines such as TNF-α and inducible nitric oxide synthase (iNOS), whereas type 2 macrophages express anti-inflammatory cytokines such as IL-10 and arginase-1. In our mouse model of CAC, we found the presence of human MUC1 induced type 1 macrophages. This can explain the inflammatory role of MUC1 and its activity in tumor promotion and progression. 

A different landscape of infiltrating inflammatory cells in the presence of MUC1 can also be explained by the fact that hypoglycosylated MUC1 is chemotactic to inflammatory cells [91,92,93]. The short glycans on the tumor MUC1 directly bind inflammatory cells leading to tumor cell detachment from the primary tumor site and migration to a secondary site. Tumor microenvironment also influences the recruitment and differentiation of dendritic cells (DC) and their ability to prime T cells. Inability of DC to present tumor antigens to T cells is one of the mechanisms of tumor escape from immune surveillance. Hypoglycosylated MUC1 recruits immature DC and induces their aberrant maturation. In in vitro experiments, co-cultures of DC precursors with MUC1+ tumor cells inhibited their differentiation and function. In addition, DCs tend to adhere more to MUC1-STn+ cancer cells and this contact inhibits DC maturation, inducing them to produce IL-10 and reducing their ability to trigger the protective T helper 1 (Th1) response [91,94]. 

Aberrant glycosylation of MUC1 also inhibits its processing as a tumor antigen by DC and presentation to T cells. This is compounded by inefficient transport of endocytosed MUC1 from early endosomes to later endosomal compartments in DC for degradation into peptides or glycopeptides that bind to class II MHC. We have previously proposed that the exceptional avidity of binding between the tandemly repeated carbohydrates of MUC1 and mannose receptors on DC does not allow their dissociation after cellular internalization and thereby MUC1 degradation [95]. It was also observed that STn+ cancer cells inhibited generation of Th1 T cells further, limiting the ability of the anti-tumor T cell response [96]. This all affects development of stronger anti-MUC1 immunity that could otherwise target the abnormal MUC1 as an antigen on tumor cells for efficient cancer immunosurveillance.

Natural killer (NK) cells play a central role in tumor immunosurveillance [97]. However, tumor cells are capable of avoiding recognition and destruction by innate immune cells. Several studies have reported that increased expression of sialylated glycans on the surface of tumor cells promotes immuno-evasion and protects cancer cells from NK cell lysis. The interaction of NK cells with tumor sialoglycans is mediated by inhibitory receptors sialic acid-binding immunoglobulin-like lectin-(siglec)-7 and 9. After the engagement of siglecs with the sialoglycans on tumor cells, NK cell function is attenuated. By using synthetic glypolymers that mimic cell-associated glycans, Hudeck et al. showed that remodeling of the sialylation status of cancer cells affected the response of NK cells via siglec-7. Specifically, increased glycan sialylation inhibited NK cell activation [98]. These data are supported by another report that showed that treatment of tumor cells with neuraminidase that removes sialic acid, restores their sensitivity to NK cell cytotoxicity and promotes their cytokine secretion [99]. 

## 7. Conclusions

Overexpression and aberrant glycosylation of MUC1 on tumor cells influences many critical steps in tumor initiation, progression, and metastasis as well as in tumor immunosurveillance. We found that this hypoglycosylated tumor form of MUC1 is also overexpressed during chronic inflammation and in premalignant lesions that progress to tumors. Truncated sugars on MUC1, such as T, Tn, and their sialylated forms, are associated with more advanced tumor stages and poor prognosis. Shorter *O*-glycans confer oncogenic properties to MUC1 leading to aberrant intracellular signaling that results in increased transcription of several target genes including those encoding pro-inflammatory cytokines. Secretion of pro-inflammatory cytokines enhances the recruitment of inflammatory cells into the tumor site. Inflammatory cells, including macrophages and DC, are a further source of inflammatory cytokines, which in turn promote tumor progression and metastasis (Figure 2). Therefore, even if MUC1 is not a direct initiator of neoplastic transformation, it plays a key role in aggravating inflammatory conditions that create a pro-tumor microenvironment, accelerating tumor growth and metastasis. In addition, hypoglycosylated MUC1 can inhibit its own processing by DC as a tumor antigen and presentation to T cells, thereby preventing anti-tumor immune responses. Thus, targeting hypoglycosylated MUC1 with various drugs or biologics that can modulate or inhibit some of the many functions we described above may lead to inhibition of tumor growth and promotion of anti-tumor immune responses, including anti-MUC1 immunity. Vaccines targeting the hypoglycosylated MUC1 are one modality being developed by us and others in an effort to induce antibodies and T cells that can eliminate inflammation and/or tumor initiating cells that express this form of MUC1, thereby preventing further inflammation and promoting anti-tumor activity of many effector cells in the microenvironment.
